# Barriers and facilitators to health care access for people experiencing homelessness in four European countries: an exploratory qualitative study

**DOI:** 10.1186/s12939-023-02011-4

**Published:** 2023-10-06

**Authors:** Christina Carmichael, Tobias Schiffler, Lee Smith, Maria Moudatsou, Ioanna Tabaki, Ascensión Doñate-Martínez, Tamara Alhambra-Borrás, Matina Kouvari, Pania Karnaki, Alejandro Gil-Salmeron, Igor Grabovac

**Affiliations:** 1 Centre for Health, Performance and Wellbeing, Anglia Ruskin University, East Rd, Cambridge, CB1 1PT UK; 2 School of Psychology, University of East Anglia, Norwich Research Park, Norwich, NR4 7TJ UK; 3 Centre for Public Health, Department of Social and Preventive Medicine, Medical University of Vienna, Kinderspitalgasse 15, Vienna, 1090 Austria; 4 PRAKSIS – Programs of Development, Social Support and Medical Cooperation, Stournari 57, Athens, 104 32 Greece; 5 Polibienestar Research Institute, University of Valencia, Carrer del Serpis, 29, Valencia, 46022 Spain; 6 Environmental and Occupational Health, PROLEPSIS – Institute of Preventive Medicine, Fragoklisias street 7, Athens, 151 25 Greece; 7 International Foundation for Integrated Care, Linton Road, Oxford, OX2 6UD UK; 8 International University of Valencia, Calle del Pintor Sorolla, 21, Valencia, 46002 Spain

**Keywords:** Homelessness, Primary health care, Health care disparities, Qualitative research

## Abstract

**Background:**

People experiencing homelessness (PEH) are known to be at higher risk of adverse health outcomes and premature mortality when compared to the housed population and often face significant barriers when attempting to access health services. This study aimed to better understand the specific health care needs of PEH and the barriers and facilitators associated with their timely and equitable access to health services in the European context.

**Methods:**

We conducted an exploratory cross-national qualitative study involving people with lived experience of homelessness and health and social care professionals in Austria, Greece, Spain, and the UK. A total of 69 semi-structured interviews comprising 15 social care professionals, 19 health care professionals, and 35 PEH were completed, transcribed, and analysed thematically.

**Results:**

Findings were organised into three overarching themes relating to the research question: (a) Health care needs of PEH, (b) Barriers to health care access, and (c) Facilitators to health care access. Overall, the general health of PEH was depicted as extremely poor, and mainstream health services were portrayed as ill-equipped to respond to the needs of this population. Adopting tailored approaches to care, especially involving trusted professionals in the delivery of care, was identified as a key strategy for overcoming existing barriers.

**Conclusions:**

The results of this study indicate there to be a high degree of consistency in the health care needs of PEH and the barriers and facilitators associated with their access to health care across the various European settings. Homelessness in itself is recognized to represent an essential social determinant of health, with PEH at risk of unequal access to health services. Changes are thus required to facilitate PEH’s access to mainstream primary care. This can also be further complemented by investment in ‘in-reach’ services and other tailored and person-centred forms of health care.

**Trial registration:**

This study was registered retrospectively on June 6, 2022, in the registry of ClinicalTrials.gov under the number NCT05406687.

**Supplementary Information:**

The online version contains supplementary material available at 10.1186/s12939-023-02011-4.

## Background

Over the last 20 years, homelessness has received increased attention as a concern for the public health community and policymakers worldwide. The relationship between homelessness and health is complex and bi-directional, with poor health known to be both a predictor and a consequence of homelessness [1, 2]. Chronic illnesses, infectious diseases, and mental health conditions have all been shown to be over-represented among people experiencing homelessness (PEH) [3], with rates of tuberculosis and hepatitis C much higher when compared with people living in secure housing [4, 5]. PEH are subsequently at higher risk of premature mortality and have a lower life expectancy than the housed population [6].

Poor health among PEH may be partially explained by a high prevalence of environmental and behavioural risk factors among them. However, a growing body of literature has also drawn attention to the barriers encountered by PEH when attempting to access and navigate what are often complex and fragmented health care systems [7, 8]. It has been found that PEH are less likely to utilise mainstream primary care services [9, 10]; that is, those which are available to the general population through ‘standard’ primary care pathways and not specifically tailored to meet the complex needs of marginalised communities [11]. Instead, evidence suggests that PEH often rely on acute health care services such as emergency departments in hospitals [3, 12, 13]. As a result, previous studies have indicated that health-related issues often go untreated within this population, and diagnosis of severe conditions may be delayed [1], while in emergency admissions, long-term conditions are often managed in crises leading to more readmissions [14].

Within this context, there is growing recognition that the health outcomes of PEH should be understood through the framework of social determinants [1, 15], emphasizing the structural and societal factors that advantage or disadvantage specific populations and shape individuals’ access to and experiences of health care. Housing status, for example, has been clearly identified as a social determinant of health by the World Health Organisation [16]. Homelessness is also known to intersect with other forms of ‘deep social exclusion’, including psychological trauma, unemployment, food insecurity, substance use and experiences of institutional care – each of which has the potential to further exacerbate health inequalities [17].

The need to account for these social determinants of health and improve the inclusivity of health care services for socially excluded populations has been a named priority in the European context and beyond [13, 18]. The pressing need to improve the effectiveness of health care provision for PEH, in particular, is further reinforced when we look at the current scale of homelessness across Europe. While inconsistencies in definition and measurement make estimating the exact number of PEH difficult, the sharp upward trend across almost all EU member states and the UK over the last decade is irrefutable [19, 20].

Exploring how PEH explain their own needs, preferences, and concerns around health is essential for developing meaningful person-centred interventions and improving engagement with primary care and preventative services [21]. As well as this, we must document examples of best practices already in place so that these may be replicated more widely. While PEH and frontline professionals often have a significant understanding of ‘what works’, this knowledge is frequently overlooked within health care policy and research discourses [22].

With this in mind, this qualitative study aimed to better understand the health care needs of PEH and the barriers and facilitators that prevent and enable their access to health care services from the perspective of those ‘on the ground’ – that is, people with direct experience of homelessness, staff working in homelessness-related support services and frontline health and social care professionals -in four European countries. Of the different health care systems in the countries included, all follow a model of either universal health insurance (Spain and the UK), service coverage (Greece), or nearly universal coverage for health care (Austria), financed through taxation or social insurance schemes [23]. While there are a small number of existing qualitative studies on this topic, these have tended to focus specifically on a single locality [24, 25]. This qualitative study is therefore understood as relatively novel in its scale and use of cross-national data and its inclusion of PEH, social care professionals and health care professionals together within one study. It sits within a larger-scale project funded by the European Commission’s Horizon 2020 Programme entitled “Cancer prevention and early detection among the homeless population in Europe: Co-adapting and implementing the Health Navigator Model” (CANCERLESS). CANCERLESS aims to develop, pilot, and evaluate a community-based intervention to improve primary and secondary cancer prevention among PEH in the four countries named above.

## Methods

### Study design

This study employed a qualitative research design, with data collected through a series of semi-structured interviews conducted by members of the research team in field settings across the four partner countries (Austria, Greece, Spain, and the UK) between August and October 2021. The choice to adopt a qualitative research design reflected the exploratory aims of the study and ensured that participants were offered a level of freedom to share what they felt to be most important or relevant. Given the sensitive nature of the research topic, the non-prescriptive nature of qualitative interviews was deemed most appropriate as they allow for rapport-building and increase the likelihood that participants feel comfortable disclosing personal experiences and opinions [26].

### Data collection

In each of the four countries, PEH were recruited with the assistance of a specific health and social care professional or homelessness organisation with whom an established partnership had already been established for the purposes of the CANCERLESS project. Each of these organisations works directly with a population of PEH, meaning that staff representatives were able to assist in making appropriate introductions between researchers and potential participants. Participants were defined as experiencing homelessness if their living situation corresponded with the European typology of homelessness and housing exclusion [27]. This typology encompasses a range of circumstances, including literal rooflessness, living within institutions, services, or shelters, and living in insecure or inadequate housing. All participants were required to be over the age of 18 and be able to provide informed consent.

Professional participants were also drawn from these partner organisations, combined with an element of ‘snowball’ sampling across professionals. The professional participant group encompassed two groups: health care professionals (including nurses, oncologists, psychiatrists, and primary care physicians/general practitioners) and psycho-social professionals working in public and voluntary sector organisations (including support workers and social workers). Research teams utilised local knowledge in identifying which professionals would be most appropriate depending on how service provision operated in the specific setting. In some cases, there were established relationships between the professional participants and the PEH included in the study – for example, where both members of staff and service users were interviewed within the same organisation. However, information pertaining to specific relationships between participants was not recorded nor required for participation.

Interviews took place within the partner organisations, in either private or semi-private settings (e.g. an office or a quiet area of a communal space) depending on the participants’ preferences and were conducted by researchers experienced in qualitative interviewing and research with vulnerable populations. Topic schedules were used to guide the interviews and encourage a level of consistency in the cross-national data collection. These were developed in consultation with the staff of a homelessness organization to ensure the suitability of language and content. Specific topic schedules were tailored to PEH and professional participant groups and included questions on the perceived health of PEH and the barriers and facilitators associated with accessing health care (see Appendix 1).

### Data analysis

Interviews were audio-recorded and transcribed verbatim in their respective languages, either manually or using assistive software. Transcripts were analysed iteratively using the guidelines for thematic analysis set out by Saldaña [28] and aided by either NVivo v12 or Atlas.ti v8 software.

In the first cycle of analysis, researchers in each country worked through their respective datasets by systematically attaching codes to capture meaning in the text on a line-by-line basis. Here, using an inductive approach ensured that the participants’ language, perspectives, and priorities were foregrounded in the analytical process. In the second coding cycle, researchers in each country reviewed, merged, and condensed initial codes to further focus their analysis [28]. Based on the outcomes of coding, a thematic framework was developed by the first author which was used by the teams in each country to proceed with their country-specific analyses. Then, the work was finalised through ongoing discussion and collaboration with the entire research team. This consisted of a series of tentative themes that aimed to best capture the outcomes of the focused coding. These themes were then organised under three main headings reflective of the study aims (Health care needs of PEH, Barriers to health care access, and Facilitators to health care access). Working through and structuring the data in this way allowed the researchers to remain open to unexpected lines of inquiry whilst maintaining a focus on the specific aims of the study. Although coding was completed on a country-by-country basis, researchers met regularly to discuss the data analysis, and the themes presented in this paper are reflective of the full dataset; where particular points of difference were identified in the data, these have been noted in the Results section.

Data were analysed in each respective language, but illustrative quotations have been translated into English. Where required, translation was completed by researchers with high proficiency in both the local language and English and internally checked by another member of the research team to ensure accuracy. Uncertainties or differences in interpretation were resolved through consultation with the initial translator, fostering collaborative decisions to achieve the most precise translation.

### Ethical practice

As the lead institution, the Ethics Committee of the Medical University of Vienna approved the overall study, and each partner country’s research team obtained additional approval from their designated ethical review board or institution before beginning data collection. Prior to the start of the interviews, all participants were provided with an information leaflet about the study and given the opportunity to ask questions before deciding whether they would like to participate. In all cases, participants were reminded that their participation was entirely voluntary and that they did not need to answer any questions that made them feel uncomfortable. PEH were also specifically reminded that refusal to participate would not result in any negative ramifications or hinder their access to health and social care services in any way. Informed consent was sought from and provided by all participants through the use of a signed form; this was also checked verbally at the start of the interview.

All interviews were audio-recorded and subsequently transcribed, omitting all identifiable details, with reference codes used in place of names. Throughout this paper, quotations have been carefully considered to ensure they do not reveal an inappropriate level of detail about specific participants. Data have been stored securely in line with data protection regulations.

## Results

Overall, 69 interviews were conducted across the four countries, typically lasting between 20 and 45 min. The PEH participant group comprised 35 participants, including 13 women and 22 men, with ages ranging from 25 to 71 and a mean age of 50. The reported housing circumstances of the participant group varied and included individuals experiencing literal rooflessness, those living in temporary or supported accommodation, and those staying with friends or family. All PEH were also asked to self-report any health conditions, further details of which are provided in Table 1.

**Table 1:** Participant information – People Experiencing Homelessness

ID	Location	Age	Gender	Ethnicity	Housing circumstances (ETHOS category)	Self-reported health conditions
PEH1_AT	Vienna, Austria	40	Male	Iraqi	8.1. Temporarily with family/friends	Several burns, stress
PEH2_AT	Vienna, Austria	50	Female	Georgian	8.1. Temporarily with family/friends	Occasional headaches
PEH3_AT	Vienna, Austria	56	Male	Austrian	2.1. Night shelter	Diabetes, leg pain
PEH4_AT	Vienna, Austria	29	Male	Arabic	8.1. Temporarily with family/friends	No health issues reported
PEH5_AT	Vienna, Austria	47	Male	Serbian	7.2. Supported accommodation for formerly homeless people	Respiratory/lung problems
PEH6_AT	Vienna, Austria	42	Male	Bangladeshi	8.2. No legal (sub)tenancy	Hip pain
PEH7_AT	Vienna, Austria	55	Male	Austrian	11.3. Temporary structure	No health issues reported
PEH8_AT	Vienna, Austria	54	Female	Slovakian	11.3. Temporary structure	Cervical carcinoma
PEH1_GR	Athens, Greece	56	Male	Pakistani	13.1. Highest national norm of overcrowding	Hepatitis C, psoriasis, cirrhosis of the liver, ophthalmological and orthopaedic problems
PEH2_GR	Athens, Greece	56	Male	Greek	1.1. Public space or external space	Regurgitation, gastritis, gallstones in the gallbladder
PEH3_GR	Athens, Greece	32	Female	Congolese (DRC)	11.2. Non-conventional building	Menstrual cycle abnormalities, uterine cancer under investigation
PEH4_GR	Athens, Greece	49	Female	Congolese (DRC)	8.1. Temporarily with family/friends	Cardiac blood pressure, gastritis
PEH5_GR	Piraeus, Greece	61	Male	Greek	1.1. Public space or external space	Diabetes, cardiological issues
PEH6_GR	Piraeus, Greece	58	Male	Greek	3.2. Temporary accommodation	Skin cancer
PEH7_GR	Athens, Greece	71	Female	Greek	3.2. Temporary accommodation	Asthma
PEH8_GR	Piraeus, Greece	62	Female	Finnish	11.2. Non-conventional building	Oesophageal cancer, recovered from anal cancer, problem in the spinal column
PEH9_GR	Athens, Greece	57	Male	Iranian	1.1. Public space or external space	Depression
PEH10_GR	Athens, Greece	60	Male	Greek	3.1. Homeless hostel	No health issues reported
PEH1_ES	Madrid, Spain	65	Male	Spanish	3.1. Homeless hostel	Non-specified cancer, inguinal hernia, vision problems in the right eye, former alcoholic, former smoker
PEH2_ES	Madrid, Spain	56	Male	Spanish	3.1. Homeless hostel	Wheelchair-bound, leg orthosis, diabetic foot
PEH3_ES	Madrid, Spain	51	Male	Spanish	3.1. Homeless hostel	Post-COVID19, leg pain
PEH4_ES	Madrid, Spain	51	Male	Spanish	3.1. Homeless hostel	Severe heart disease, heart surgery, chronic high cholesterol
PEH5_ES	Madrid, Spain	50	Female	Bolivian	3.1. Homeless hostel	Former cancer patient (colon), diabetes
PEH6_ES	Madrid, Spain	52	Female	Spanish	3.1. Homeless hostel	Recovering from a fractured tibia and fibula, hypertension, historical substance addiction – currently in relapse
PEH7_ES	Madrid, Spain	59	Male	Spanish	3.1. Homeless hostel	Reduced mobility, chronic pain
PEH8_ES	Madrid, Spain	49	Female	Moroccan	3.1. Homeless hostel	Breast cancer, hypertension, high cholesterol
PEH9_ES	Madrid, Spain	61	Male	Senegalese	3.1. Homeless hostel	Post-COVID19, respiratory problems, high blood pressure
PEH10_ES	Madrid, Spain	49	Female	Uruguayan	3.1. Homeless hostel	On crutches, thyroid condition, reduced mobility, spinal problems, fibromyalgia
PEH11_ES	Madrid, Spain	48	Female	Spanish	3.1. Homeless hostel	Bone metastases, asthma
PEH1_UK	Norfolk, UK	25	Male	British	7.2. Supported accommodation for formerly homeless people	Epilepsy
PEH2_UK	Norfolk, UK	34	Male	British	7.2. Supported accommodation for formerly homeless people	No health issues reported
PEH3_UK	Norfolk, UK	42	Male	British	3.1. Homeless hostel	Multiple mental health conditions
PEH4_UK	Norfolk, UK	45	Female	British	Own tenancy (formerly 3.1. Homeless hostel)	Multiple mental health conditions
PEH5_UK	Norfolk, UK	42	Female	British	8.1. Temporarily with family/friends	Emphysema, kidney issues, chronic pain
PEH6_UK	Norfolk, UK	39	Male	British	7.2. Supported accommodation for formerly homeless people	Lung cancer

The professional participant group included 15 professionals working in social care and homelessness support services and 19 health care professionals, including primary care physicians, oncologists, nurses, and psychologists. Details of the professional participant group are provided in Table 2.

**Table 2:** Participant information – Health and Social Care Professionals

Location	Job role	n (%)
Vienna, Austria	General practitioner	5 (15)
	Social worker	4 (12)
	Oncologist	1 (3)
	Support worker	1 (3)
Athens, Greece	Child and adolescent psychiatrist	1 (3)
	Nurse	1 (3)
	Oncologist	1 (3)
	Patients’ organisation professional	1 (3)
	Primary care practitioner	1 (3)
	Psychiatrist	1 (3)
	Social worker	1 (3)
	Sociologist	1 (3)
Madrid, Spain	Nurse	2 (6)
	Primary care practitioner	2 (6)
	Social worker	2 (6)
East of England, UK	Nurse	3 (9)
	Primary care practitioner	1 (3)
	Support worker	5 (15)

### Health care needs of PEH

#### Prevalence of poor health among PEH

The self-reported data from PEH (Table 1) indicated a high prevalence of both physical health conditions and chronic illnesses and this was further reiterated through the interview accounts across all settings:

“*Respiratory issues, vascular issues, trench foot issues … that’s the majority of the people that I see … A lot of bacterial endocarditis … and a lot of bacterial neuropathy, from sleeping rough. Things like Hepatitis C, a lot of people have.*” (Nurse, UK).

“*People who have problems with alcohol and heart diseases usually come here to the centre. There are a lot of liver diseases, poorly controlled diabetes, and associated problems such as amputations and vision loss.“* (PEH5_ES).

Mental ill health was also recognised as being extremely commonplace within the homeless population. Many of the PEH explained that they were diagnosed with a mental health condition or multiple co-occurring mental health conditions. In contrast, others alluded to mental ill health without mention of any formal diagnosis:

*“It was really, really bad, not so much anything physical, just me in myself … I was sleeping rough, and yeah, wasn’t looking after myself, didn’t have any interest in anything at all.”* (PEH2_UK).

This was reiterated by social care professionals who highlighted the prevalence of undiagnosed mental health issues amongst the service users they worked with and that mental ill health was often interwoven with harmful substance use and addictive disorders, which raised further health concerns:

*“If they’re injecting, ulcerated legs do come up quite a lot, and they can get infected … Yeah, addiction, alcoholism, mixing their prescribed medication with alcohol, that’s a big thing.” (Support Worker, UK)*.

However, many of the professional participants were also keen to highlight the heterogeneous nature of the homeless population and the wide range of health profiles they encountered in their work. Here, it was felt that PEH’s health care needs depended on how and whether an individual’s experience of homelessness was interwoven with other forms of vulnerability and social exclusion (for example, migrant status, harmful substance use, and experience of institutionalisation). Notably, this heterogeneity was explained as creating additional challenges for health and social care services as a ‘one size fits all’ approach was rarely suitable:

“*The homeless population is a population that has rather mixed characteristics … some people that are homeless due to economic reasons, there are some people that are homeless as a result of the use of drugs, with dependency, and there are some homeless people with severe mental illness … they have very different health needs.*” (Psychologist, Greece).

### Impact of living conditions

PEH consistently described how their living conditions while experiencing homelessness had directly impacted their physical and mental health and resulted in the development of new health issues and the exacerbation of pre-existing conditions. Factors cited included the poor quality of accommodation, the lack of sleep, the lack of nutritious meals, and limited access to personal hygiene facilities. The social isolation and loneliness associated with being homeless were also explained as having a direct negative impact on PEH’s mental health and increased likelihood of harmful substance use:

*“It’s very hard not to have a place to live … People who live on the street can catch a lot more diseases, you know, due to not having good health, due to not having a clean home.”* (PEH3_ES).

*“Absolutely, homelessness does [have an impact on health]. Not only on mental health but also physical health. Because people are constantly moving. Whether it’s cold, warm, or sub-zero, they’re constantly outside and of course, that affects the body. … When something hurts, many people solve it with alcohol.”* (PEH5_AT).

From the professional perspective, the impact of homelessness on health was recognised as being particularly pronounced for those with pre-existing and chronic physical health conditions, which are often reported to have worsened due to the living conditions and lifestyles associated with homelessness. For example, several professionals explained that PEH often struggle to self-manage their diet or medication whilst experiencing homelessness:

*“Their eating habits on the street are basically whatever they can get their hands on. So, let’s say a diabetic, a heart patient or a person with high cholesterol… they may not have access to food that is beneficial to their condition.”* (Psychologist, Greece).

### Barriers to health care access

#### Support needs of PEH

Across all settings, it was recognized that the extent of poor health, and particularly mental ill health, among PEH, was in itself a barrier to accessing health care. This was explained in terms of the inability of mainstream health services to respond appropriately to patients presenting with complex support needs, but also in terms of the capacity of individuals to take active steps to engage with services:

“*People are mentally ill, and their conditions are not treated, but it is not that they are not treated because there is no help available, but because people are simply so ill that they cannot accept any help*.” (Social Worker, Austria).

Several participants also noted that PEH could struggle with social interactions and may face difficulties when attempting to communicate their needs to unfamiliar professionals:

“*Sometimes I’ve thought … are they understanding me? Sometimes I’ve got nervous, my mind goes blank, I get really confused … it’s just really stressful.*” (PEH4_UK).

Language barriers were described as posing a major challenge in Austria and Greece, reflecting the significant numbers of migrants and refugees experiencing homelessness within these settings. For example, several health care professionals in Austria spoke of being forced to enlist cleaning staff and children to facilitate communication with homeless patients, leading to inappropriate diagnosis and care. Participants also reported similar situations in Greece, where the availability of interpreters within public hospitals was described as “non-existent”.

### Preventative health care as ‘non-priority’

Participants explained that for PEH – and especially for those experiencing literal rooflessness – engagement with preventative and primary health care is not generally treated as a priority. Instead, the time and energy of PEH were seen to be taken up with meeting more immediate and ‘basic’ needs, such as accessing shelter, food, substances, and welfare benefits. Notably, and across the participant group, meeting these sorts of basic needs did not appear to be conceptualised as a *part* of or a form of health care, but instead as something that prevented health care from taking place:

“*The priorities of a person living in a house can be taking care of themselves, going to the doctor … but when you are living on the street, your priority is to eat something and not have your belongings stolen*.“ (PEH3_ES).

*“Homeless people worry about their health when they are already very sick … but normally they worry more about drugs, food … their priorities are very different from the rest of the people*.“ (N2_ES).

From the perspective of several of the professionals, the perceived barrier was not only that PEH were faced with these competing demands but that, in some cases, their understanding of how to self-manage their health and recognise potential symptoms was also very limited:

*“Because they are faced with so many other problems in their life, sometimes they don’t even realise that there is a problem, or even if they do, they don’t really realise the need to address that.”* (Primary Care Physician, UK).

As a result of this, health care professionals working in mainstream services explained that by the point that they had actually engaged with PEH, the extent of their condition had often worsened to the point that primary or preventative services were no longer the most appropriate option for care, and instead intervention by acute health services was required:

“*[Health-related issues] are usually left until things have got a lot worse … So when we do see chest infections, they are well-established, and when we see wounds, they are well-established, and when we see people with drug and alcohol issues, their addictive behaviours are well-established.”* (Nurse, UK).

### Social stigma

While some accounts included examples of positive relationships between PEH and health care professionals, the majority of participants agreed that PEH were generally treated poorly in comparison to the general public, with many of the professional participants perceiving PEH to be actively discriminated against. For example, several secondary accounts provided by social care professionals recalled instances where PEH had been turned away from health services due to the way they looked or presented or where staff had been rude or dismissive:

*“Some of them have not even been allowed into GP surgeries because they do not correspond to the ideal patient image of ‘freshly showered’, ‘comes to the appointment well-groomed and on time’, ‘has everything you need’, but are perhaps not freshly washed, are perhaps slightly drunk or more heavily drunk at times, are perhaps loud, are perhaps unpleasant … [they] notice that they are looked at strangely, that they are either not treated or are treated much later.”* (Social Worker, Austria).

Here, it was also reported that health care professionals would often make assumptions about the health needs and required treatment of PEH, specifically in terms of presumed substance abuse. The potential that health care professionals were disregarding symptoms of severe health conditions was raised as a specific concern:

*“It’s a real second-class citizen system for homeless people … There’s an expectation that the patient is usually more needy and drug seeking. So, every presentation usually comes back to a behaviour or addiction illness as the diagnosis, rather than pursuing it.”* (Nurse, UK).

From the perspective of PEH, the fear of mistreatment, and a desire to preserve and protect their dignity, were described as actively dissuading them from attending mainstream health care services in their local area. It was also that, for some PEH, there also existed an element of self-stigma, whereby feelings of shame about their circumstances had been internalised and further deterred them from engaging with health care services:

*“The main barrier [in accessing health care services] is unfortunately created by us, the people who are homeless because it is very shameful to say that I have no home.“* (PEH11_ES).

### Inflexible and fragmented systems

Across all settings, the inflexible and often fragmented nature of health care systems was identified as a key barrier to timely access to health care for PEH. In particular, the need to attend various appointments or services located in different settings was described as being incompatible with the chaotic and often transient lifestyles of PEH. As a result, from the point of view of primary care physicians, there was felt to be minimal opportunity for implementing continuity of care:

*“At the time of follow-up, a homeless person is seen as “uncomfortable” for us … I say this because we will not be able to have a follow-up, and there may be difficulties in coordinating with social workers, firstly because there is not always one in a health care centre, or because you have to refer them [the homeless patient] to another place and you don’t know if that person is going to attend for any reason.“* (Primary Care Physician, Spain).

Expressly, in the UK context, participants also indicated that mainstream health services generally have a deficient level of tolerance towards missed appointments and ‘non-standard’ behaviours (for example, style of language). In some cases, this resulted in homeless patients being excluded from services or facing an extended wait for care:

“*Rather than saying ‘I’ve been waiting two hours’, they say ‘I’ve been waiting two fucking hours’ and that’s then classed as verbal aggression and then they’re removed for the sake of a little bit of street vernacular.”* (Nurse, UK).

In a similar vein, professionals in the Greek context explained that PEH would often only be seen by health services when accompanied by a homelessness or social care professional, thus reducing their opportunities to access health care promptly:

*“[Health services] were saying ‘he’s on his own so we can’t care for him, he needs to have an assistant’”* (Primary Care Physician, Greece).

### Practical and financial barriers to health care access

The lack of flexibility within mainstream health systems was also made apparent in the many examples of practical and bureaucratic barriers that PEH had faced when attempting to access local health services. Several of the participants experiencing homelessness recalled that they had been refused access to primary care services or denied prescription medication because of a lack of fixed address or photo identification and had struggled to navigate appointment booking systems without a phone or email address:

*“Most of the time, they say they will send the information to your mobile, but you do not have a mobile … or they will send the letter to your house, but you do not have a house”* (PEH8_ES).

Participants in Austria, Greece, and Spain also explained that a central barrier was the absence of health insurance or appropriate documentation (i.e., a health card) among PEH, which was effectively excluding them from accessing mainstream health and social care systems. Indeed, while some alternative services for uninsured persons were mentioned, professional participants in these settings emphasized significant gaps in the framework of provision. This issue was described as being particularly problematic for migrants and refugees experiencing homelessness, where instances of being turned away from health services were common.

### Resource scarcity

Finally, participants consistently explained that many of these other barriers exist and should be understood against a backdrop of resource scarcity within European health care systems, which has further intensified since the start of the COVID-19 pandemic. This was particularly pronounced in Spain, Greece, and the UK, where long waiting periods for appointments and treatment were commonly vocalised as a source of frustration for PEH. While this as a barrier is not unique to PEH, it seemed from the accounts that this was having a notably negative effect on individuals already in critical circumstances. Indeed, there was a palpable sense of defeat and hopelessness among some participants who were seeing their health worsen, but at the same time, struggling to access the care they needed:

“*Many months have gone by, and my condition remains the same. I am not being seen. And when the time comes to do the fourth test, because I feel that my condition is getting worse, I will be told to do the other three tests all over again.”* (PEH3_GR).

*“A few days ago, I was going up the wall, I was hysterical … Then I called my doctor and told him, and he told me that he was going to send me to the psychologist … They gave me an appointment on December 23rd, and we are currently at the beginning of September … the length of time between the moment when you have the problem and when they attend to you is absurd.”* (PEH10_ES).

### Facilitators to health care access

#### Positive patient-professional relationships

While most experiences of accessing health care were described in negative terms, several of the PEH singled out a specific professional or professionals – for example, their local primary care physician, nurse, or social worker – with whom they had developed a positive and trusting relationship. Where this was the case, it was made clear that these relationships had helped to encourage these PEH to engage with health care services and made them feel more comfortable about the prospect of continuing to do so in the future. When asked what they appreciated about these relationships, the importance of familiarity, transparency, continuity, and availability were all emphasised:

*“There was this social worker … I kept her, only her, I sent away all the others who came. She came and found me at my spot, but instead of canned food or bread or food, she left a thermometer, painkillers, and Niflamol for my teeth. She listened to me. It was not about what she gave me, but that she listened to me.”* (PEH2_GR).

“*They [health care professionals] were very clear with me from the beginning, they care about telling me about everything that is happening to me.*” (PEH3_ES).

### Tailored approaches to service delivery

Participants repeatedly emphasized the value of adopting tailored and person-centred approaches to health care delivery and spoke positively about services that operated in ways that were responsive to the needs of PEH. These included examples of in-reach, whereby health care professionals visited and operated within homelessness shelters and day centres; services that did not require pre-booked appointments but instead allowed patients to ‘drop in’ as they needed; and mobile health care units which moved around the local area and provided easy-to-access care:

*“The most important thing is that the contact [with PEH] is direct … one must approach the affected persons directly. Also, offer continuous care or treatment by way of a low-threshold approach.”* (Primary Care Physician, Austria).

“*The service [mobile health care unit] was there, and they [health care professionals] offer it to you … you don’t have to go looking*.” (PEH5_UK).

In both Greece and the UK, examples were also provided of health care being delivered less formally, for example, by adapting the language used by professionals or by including someone with lived experience of homelessness in the appointment. These sorts of approaches were felt to reduce feelings of anxiety and intimidation that many PEH associate with mainstream health services:

“*When I talk to drug and alcohol users, I always use the street terminology … and I dress like this for a reason, I think uniforms are a barrier … if I’m wearing a uniform, people won’t engage.”* (Nurse, UK).

“*Our best practice was exactly this … having a person who belongs to or used to belong to this group. It is fundamental for them to feel that a person is like them, understands them, or “speaks their language”, their slang. This makes communicating with them much easier.”* (Nurse, Greece).

### Involvement of non-clinical professionals

Linked both to the need for tailored approaches to care and the importance of trusting relationships, participants repeatedly indicated that attempts to engage PEH in health care were most successful when mediated by professionals working in social care and homelessness support services who understand the needs of this population and with whom PEH were already familiar. The involvement of support staff was felt to increase the confidence of PEH to address and manage their health and improve adherence in attending appointments, as well as promote understanding of the needs of homeless patients among health care professionals:

“*We’ve got one young lady, she’s terrified of the doctor, and she just wants you there, she’ll say, ‘please hold my hand,’ and then she’ll talk to the doctor because you’re with her … I know how to talk to them to encourage them to tell the doctor the symptoms.”* (Support Worker, UK).

However, in the Austrian setting, it was also emphasized that there should not be this level of reliance on social and support workers. Instead, health services should take steps to ensure that PEH can access affordable health care whether or not they are engaged with non-clinical professionals:

“*For me, the most burning point is that there should be health care for all, and we have to be serious about it. Not only on paper and in theory but also the actual design of the services. This means that the services have to be implemented in such a way that people can make use of them even if they have no one to take them by the hand.”* (Primary Care Physician, Austria).

## Discussion

This paper has provided an exploratory qualitative account of the perceived health care needs of PEH across four European settings and barriers and facilitators associated with their timely access to health care. A series of themes were generated from the data that best captured and summarised professionals’ and PEHs’ perspectives and experiences.

Across all of the settings included in the study, and consistent with existing literature, the general health of PEH was portrayed as being very poor [25, 29]. Many of the PEH included in the study suffered from chronic physical health conditions, mental ill health, and substance-related disorders, and these were often described as being either exacerbated by or a direct product of their experience of homelessness [1, 3]. For example, poor mental health outcomes were often explicitly attributed to the social isolation and exclusion that comes with the experience of homelessness, which is recognized elsewhere to be a direct indicator of harmful substance use [30, 31]. It was also explained that preventative ‘longer-term’ forms of health care rarely formed a priority for PEH, with their focus instead placed on meeting more immediate needs. As such, health-related issues and conditions were described as often being neglected until the point that primary health care was no longer appropriate and attendance at acute services was required [32].

It is, therefore, of genuine concern that our findings indicated that PEH face an extensive range of barriers when seeking access to timely health care. Taken together, these barriers suggest that mainstream primary health care services are often not sensitised to respond to PEH and, at times, operate with rigid procedures that are entirely compatible with the lifestyles and needs of members of this population. In line with comparable studies, this was seen through a lack of flexibility and tolerance around appointment structures and unconventional behaviours [33], the need to provide identification or have a fixed address to access health care services, and the use of systems that operate on the assumption that patients will have phone or internet access [34]. In some European settings, there exist major additional barriers for uninsured migrants who, as the findings of this study indicate, can find themselves denied access to basic health services even in instances of emergency [35] and face language barriers that have the potential to hinder the quality of their care [34]. For example, a study conducted in Spain found that migrants experiencing homelessness had higher rates of being uninsured, faced more barriers to health care access, and visited primary care and hospital services less frequently [36]. The importance of taking account of the interaction between homelessness and migrant status is a notable finding from this study and an essential recognition for health care service provision. Indeed, in a recent review of the health care experiences of homeless migrants, it was found that while harmful and discriminatory experiences can serve to dissuade the general homeless population from attending health services, this problem is particularly intensified for those who also have a migration history [37].

A particularly notable barrier across all settings was the perceived presence of stigmatized attitudes towards PEH and resultant instances of discrimination on the part of health care professionals working in mainstream service provision. This mirrors findings from a number of other recent studies, which have also highlighted how direct experiences of stigma, or even the expectation of being treated poorly in health care services, can serve to further deter PEH from engaging with health care services [35, 38]. To address this, additional training and resources for frontline health care professionals aimed at improving their understanding of the health needs of PEH are urgently needed [24, 38]. In particular, increasing the ability of professionals working in mainstream health services to facilitate signposting and linkage to wider health providers has the potential to reduce fragmentation within service provision and thus improve continuity in care [25]. A better understanding of homelessness among health care professionals would also likely allow them to build more trusting relationships with PEH and subsequently support their maintained engagement and continuity in their care.

The multitude of difficulties around access to health care was recognized as being further compounded by the broader socio-economic context, in which many European health care systems are under-resourced and under-staffed – an issue that has further intensified during the COVID-19 pandemic [39–41]. There was a clear sense in the data that issues such as long waiting times and high thresholds for access were serving to intensify feelings of hopelessness and isolation among PEH and risking their disengagement from health care services entirely. At a national policy level, one potential strategy to address this would be to allocate a specific proportion of statutory funding or GDP to health care programmes for PEH [3].

Although the overall findings of this study indicate that experiences of accessing health care for PEH are often problematic, participants did recall several examples of facilitatory practice already in place. Here, the data’s most consistent theme was the importance of trusting and empowering relationships between PEH and health care professionals. Indeed, previous research has indicated that PEH often face difficulties with social interactions and thus can struggle in communicating their personal needs to unfamiliar health care professionals [42]. The pivotal role played by professionals working in homelessness support services, who often mediate PEH’s access to health care, was also consistently evident across the participants’ accounts [43, 44], again reaffirming the importance of trust and familiarity in delivering health care to this population [42]. This suggests that using multidisciplinary teams containing professionals or peers with a specific understanding of homelessness is a promising approach in this context [45, 46].

While improving access to mainstream health care services should be a priority, the examples of best practices set out by participants in this study also indicated the value of complementing this with tailored approaches to health care delivery explicitly aimed at PEH. Examples of this include the use of ‘walk-in’ centres, in-reach services, and mobile health care units. Although these sorts of alternative services have been evidenced to combat many of the barriers noted above [42, 47], they are seemingly sporadic in both their coverage and longevity and are, therefore, not routinely available to all PEH and instead dependent on what are often short-term local initiatives [24]. As such, this finding is indicative of the need for more cohesive national and cross-national policies regarding the provision of health care for PEH, whereby examples of best practices may be shared and scaled up [38].

This study’s findings reinforce that homelessness must be consistently acknowledged as an essential social determinant of health; as Stafford and Wood [1] have previously asserted, addressing homelessness is “an important form of health care, not a separate ‘non-health’ issue”. While this has long been recognised in academic discourses, public health communities, and by organisations advocating for underserved populations, it does not consistently translate into policy agendas in the European context; the issues faced by PEH – including access to housing – are treated as distinct and separate to health [1] and the scale and health profile of homelessness is not well-monitored [48]. Moreover, the results of this study indicate that this disconnect also exists at a level of health care systems, evidenced by (a) health care services being unprepared to meet the needs of PEH; (b) the discrimination experienced by PEH when attending health care services, and (c) the conceptualisation of health care as being distinct from PEH’s ‘other’ needs, such as shelter [15].

Fundamentally, what is most needed are further measures aimed at intervening and preventing homelessness where a person is at risk and facilitating quicker access to secure accommodation where a person does become homeless. Therefore, the findings of this study align with growing calls for the implementation of Housing First (HF) approaches across Europe, whereby PEH are provided with rapid and non-conditional housing, which allows them the space and security to address other issues they may be facing [49, 50]. While specific evaluations of the health-related outcomes associated with HF have been limited to date, evidence has shown that the HF model is associated with a reduction in non-routine use of health care services, which could indicate a general improvement in health [51]. As stated above, and although the value of this approach is now widely accepted among academic and non-governmental audiences, the extent of political and financial support for the implementation of HF remains varied across Europe [52].

### Strengths and limitations

The exploratory qualitative design is an evident strength of this study as it allowed for a thorough investigation of the perceptions, experiences, and priorities of the participants of the study, including PEH, who are seldom heard within policy and practice dialogue. The systematic approach to analysis, along with the involvement of multiple researchers, also ensures the confirmability and credibility of the findings presented [53].

This study included a robust and highly varied selection of participants and is thought to be novel in its inclusion of participants from four different European countries. However, it is noted that in relying on homelessness support services to recruit participants, the experiences and needs of the ‘hidden’ homeless population who are not engaged with services are not fully represented in this study. Sampling was also focused predominantly on urban areas and therefore did not include the experiences of more rural homeless populations. In addition, it is recognised that in conducting interviews cross-nationally, the research teams in three of the four countries were required to translate original data into English; as a result, it is possible that some cultural meanings (for example, colloquialisms and metaphors) may have been ‘lost in translation’ [54].

Finally, the diversity in national health and care systems and homelessness service provision may mean that the findings presented here are not indicative of what is happening elsewhere. That being said, the commonality and consistency in the themes identified across the four settings are certainly notable and indicate that these findings have a high degree of transferability beyond the specific contexts where data was collected.

## Conclusion

Our findings reveal a substantial level of uniformity in the health care needs of PEH and the factors that either impede or facilitate their access to health care across diverse European contexts. As PEH are at a heightened risk of experiencing significant health disparities, a need exists for transformative changes aimed at improving this population’s access to mainstream primary health care systems in Europe. Augmenting this effort, investments should also be channelled into developing ‘in-reach’ services and other personalized, patient-centred health care approaches. In this regard, the findings of this study reaffirm that people with lived experience of homelessness and frontline professionals should be more consistently and directly involved in the design, delivery, and evaluation of health care services, as both groups hold unique insights and knowledge in terms of ‘what works’, and increasing their involvement has the potential to inform the development of more effective health-related provisions moving forward.

### Electronic supplementary material

Below is the link to the electronic supplementary material.

Supplementary Material 1

## Data Availability

The datasets used or analysed during the current study are available from the corresponding author upon reasonable request.

## List of abbreviations

HF: Housing First
PEH: People experiencing homelessness
