# Supplementary material for: Barriers and facilitators to health care access for people experiencing homelessness in four European countries: an exploratory qualitative study
Source: Int J Equity Health. 2023 Oct 6;22:206. doi: 10.1186/s12939-023-02011-4 (PMC10559410; doi:10.1186/s12939-023-02011-4)
Supplement: Supplementary file 1 — Supplementary Material 1 [file 12939_2023_2011_MOESM1_ESM.docx]

### Appendix 1. Question Schedules for Interviews with Specific Study Groups.

**Question schedule – People experiencing homelessness.**

1. Demographic questions - age, gender identity, sexual orientation, ethnicity, housing circumstances, long-term health conditions/co-morbidities.
2. How would you describe your health? What impacts, if any, do you feel that your experience of homelessness had on your health?
3. Who would you tend to speak to, or where would you seek help if you were to have a health-related issue or concern? Why do you choose to go there/to them?
4. Can you think of any particularly positive experiences of healthcare since you started to have housing difficulties? If so, what was it about the service/professional that made it a positive experience?
5. In what ways does being homeless make accessing healthcare services more difficult? Can you think of any specific barriers you have faced when seeking support for health-related issues?

- *Have you ever chosen not to access care or support (or delayed getting access to care or support) for a health-related issue, and if so why was that the case?*

1. Do have anything else you would like to add to this discussion, or any questions you would like to you ask me?

**Question schedule – Health and Social Care professionals.**

1. Could you start by briefing explaining your role, and the frequency and way in which you engage with people who are experiencing homeless?
2. From your experience, what are the typical health needs of people who are experiencing homeless?

- *What effects do you see homelessness having on health?*
- *What effects do you see homelessness having on healthcare access?*
- *What specific health issues/concerns do homeless people tend to present with?*

1. What advice would you give a homeless person you were working with if they were to present with a recognised symptom of a health-related issue? Where would you suggest they go to seek care/support?
2. What barriers/issues have you experienced, if any, when trying to support homeless people to access healthcare related services?

- *How easy do you find engaging homeless people with relevant services?*
- *How would you characterise the accessibility of relevant services in this area?*
- *What is your relationship with healthcare providers in the area?*
- *Do any examples come to mind?*

1. Have you faced any particular barriers in engaging/caring for/treating homeless people in terms of screening or treatment? (Individual, service or system related).

- *How easy/hard do you find engaging/screening/diagnosing homeless people?*
- *Do any examples come to mind?*

1. Do you have anything else you would like to add to this discussion, or any questions you would like to ask me?
